# Psychological Factors Related to Impotence as a Sexual Dysfunction in Young Men: A Literature Scan for Noteworthy Research Frameworks

**DOI:** 10.3390/clinpract12040054

**Published:** 2022-07-06

**Authors:** Valentina Ciaccio, Dina Di Giacomo

**Affiliations:** 1 Department of Life, Health and Environmental Sciences, University of L’Aquila, 67100 L’Aquila, Italy; valentina.ciaccio@graduate.univaq.it; 2 Postgraduate School on Clinical Psychology, University of L’Aquila, 67100 L’Aquila, Italy; 3 Laboratory of Clinical Psychology and Psychoncology of Life, Health and Environmental Sciences, University of L’Aquila, 67100 L’Aquila, Italy

**Keywords:** erectile dysfunction, sexual dysfunction, under years old, psychological, emotional impact, psychosexology

## Abstract

Despite the increasing interest in clinical practice in sexual dysfunction (SD) and the related relevance of psychological outcomes for young people, this area has been neglected in scientific scenarios. This study aims to draw on recent scientific findings and propose future research perspectives on the risk factors, diagnostic issues, and therapies that address SD for the under-40 age category, with particular attention paid to various psychological aspects. A literature search was conducted on studies published between March 2011 and March 2021. Anxiety, depression, and relational conflicts can both cause and affect sexual dysfunction. Psychological interventions have also proved to be important to organic causes; however, no review has found either sex education programs or multidisciplinary therapies. A multidisciplinary approach involving medical doctors and psychologists (trained in psychosexology) would improve not only the recognition of disorders through organic and psychogenic symptoms, but also treatment effectiveness.

## 1. Introduction

It is estimated that by 2025, 322 million men will suffer from erectile dysfunction (ED) [1,2]. ED is defined as the persistent inability to achieve and/or maintain a penile erection sufficient for satisfactory sexual performance [3,4]. This disorder is a sexual dysfunction (SD), defined as the inability to achieve any or all stages of sexual activity, of which, according to Helen Kaplan, five exist: desire, arousal, plateau, orgasm, and resolution, referred to by the acronym DEPOR [5]. Male SD includes hypoactive sexual desire in the desire phase, ED in the arousal phase, and premature ejaculation in the orgasm phase. These disorders may have psychogenic, organic, or a combination of causes. An initial analysis of the literature shows that psychological causes prevail in young people and that SD can in itself damage psychological health, especially during adolescence, and the discovery of one’s own sexual identity [6,7,8,9]. In this regard, the penis has always been considered a symbol of masculinity, and especially for a population approaching its first sexual experience, unrealistic images on the Web and of pornography can lead to confrontation with unattainable models [10].

Multiple organic risk factors exist, such as chronic diseases, environmental contaminants, drug toxicity, and unhealthy lifestyles [11].

Although the interest in SD in clinical practice has generally increased and more attention is being paid to the psychological sphere [10,12], few scientific protocols have focused on a psychological approach aimed at young people. Moreover, medical approaches to the disorders, which might be pharmacological or surgical, are prevalent. The literature reveals the presence of medical interventions not only related to functionality, but also to the aesthetics of the penis. In this regard, interventions can be non-invasive (including traction therapies and injection of fillers) and surgical (mostly increased penile dimensions and/or corrected penile curvature) [13,14,15].

We investigate the risk factors, diagnostic issues, and therapies that address SD in the under-40 age category and pay particular attention to various psychological aspects.

The presence or absence of psychological assessments and treatments in these articles was analyzed. Information was also obtained from psychological articles referred to in the reviews.

## 2. Materials and Methods

### 2.1. Study Design

The aim of the literature review was to examine what is known and not known and then to propose recommendations for practice and research. This review was conducted to map the literature on the diagnosis and treatment of male SD, especially ED, in adult men aged less than 40 years. The objectives were to map the focus of reviews from the past 10 years on the psychological assessment and treatment of cases of male SD and make proposals using the data reviewed.

### 2.2. Search Strategy

A systematic search of MEDLINE, via PubMed, Web of Science, and Cochrane library, was carried out using the keywords ‘erectile dysfunction’, ‘age’, ‘prevalence’, ‘sexual’, and ‘factor’ for reviews published from March 2011 to March 2021.

### 2.3. Inclusion and Exclusion Criteria

The extraction criteria applied the PICO criteria (population, intervention, comparison, and outcome) for the aims and targets. The inclusion criteria were (a) a review study design; (b) male sexual dysfunction; (c) adult age older than 40 years, (d) psychological evaluation; (e) psychological treatment; and (f) articles in the English language.

The exclusion criteria were as follows: (a) focus on disorders other than male sexual dysfunction; (b) partial or total focus on adults older than 40 years; (c) absence of psychological risk factors; (d) absence of psychological treatment; and (e) not full English language text.

### 2.4. Article Selection and Data Extraction

Two reviewers examined all titles, abstracts, and full texts of the selected scientific studies. The following information was extracted: (I) title, (II) authors, (III) journal, (IV) date of publication or submission, (V) country of first author, (VI) type of review, (VIII) disorder addressed, (IX) age of sample, (X) presence of psychological assessment, (XI) presence of psychological treatment, and (XII) language of full article.

### 2.5. Data Analyses

A descriptive analysis of the characteristics of the included studies was conducted. We described the source, first author’s country, date of publication, type of review, and topic. The topics included psychological approaches to intervention, presence or absence of educational interventions, related psychological disorders, and indications of multidisciplinary methods. The included reviews were classified into Narrative, Scoping, and Umbrella. This review was conducted in accordance with the PRISMA diagram [16].

## 3. Results

### 3.1. Search Results

The initial search returned a total of 106 articles from the database. The articles included were review studies.

After removing duplicate and inappropriate articles, 90 records were identified and reviewed for relevance in the title and abstract, excluding 61 articles at this stage.

An analysis of the full articles led to the removal of a further 23 articles, specifically for reasons of other languages (*n* = 3); age range older than 40 years (*n* = 8); no psychological assessment (*n* = 1); focus on other primary disorders (*n* = 7); and no psychological treatment (*n* = 4).

**Figure 1:**
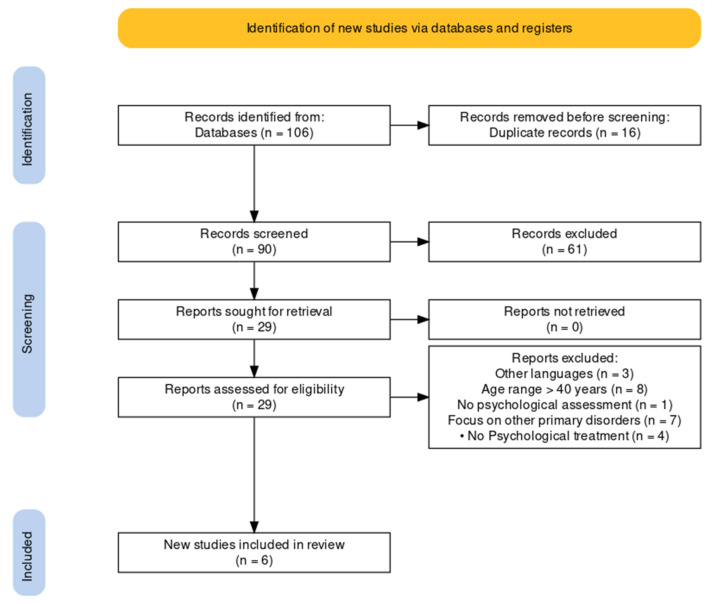
PRISMA flowchart of selection process for male sexual dysfunction review until March 2021 [17].

Figure 1 illustrates a flowchart of study selection that led to the outcome of *n* = 6 reviews.

### 3.2. Source of Articles/Studies

All articles/studies have been published in peer-reviewed journals. Only for the *Asian Journal of Andrology* (33.33%) did we find the presence of two reviews in the database; for the others, the distribution was one review per journal. Most journals (*n* = 5; 83.33%) in the final database covered andrological and sexual health (*Asian Journal of Andrology, Translational Andrology and Urology, Sexual Medicine Reviews, The Journal of Sexual Medicine*), and one (16.67%) covered continuing healthcare education (*StatPearls*). We also found no significant prevalence in the origin of the first author and the year of publication, for which two authors were from the United States (the others were from the United Kingdom, Italy, Malaysia, and Australia). Moreover, the articles were evenly distributed between 2011 and 2021, with two articles in 2017 (33.33%). The absence of a predominance of authors’ countries of origin, journals, or years highlights the worldwide nature of the subject regardless of background.

### 3.3. Type of Articles/Studies

**Figure 2:**
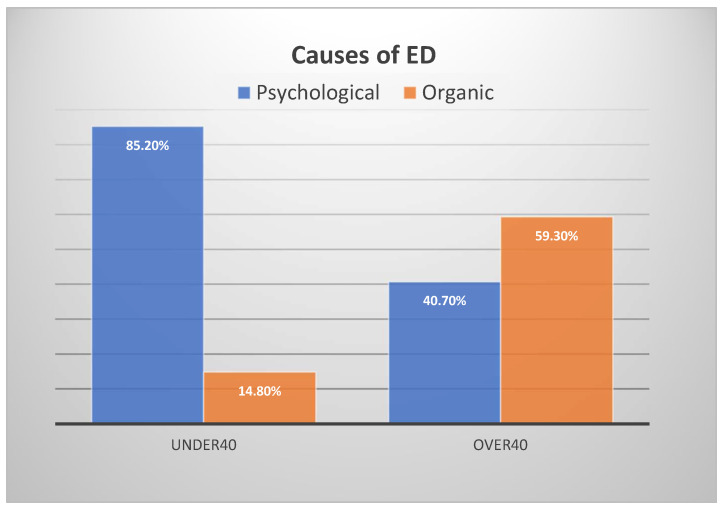
Causes of ED.

Most of the databases consisted of narrative reviews (*n* = 4, 66.67%). Almost all of the reviews focused on erectile dysfunction (*n* = 5, 83.33%), whereas only one review (16.67%) was more broadly concerned with male SD, considering ED, premature ejaculation, and hypogonadism. As shown in Table 1b, five out of six articles reported anxiety and depression as psychological factors related to ED (see Table 2 for more details on the distribution), whereas four out of six articles reported relational conditions, focusing on not only the couple, but also the family context. However, only two reviews referred to articles and/or reviews that reported studies using tests for anxiety and depression. In two other reviews, only studies on depression were reported. The last two articles had only a reference to tests administered to people over age 40 but not to our target group (Figure 2).

**Figure 3:**
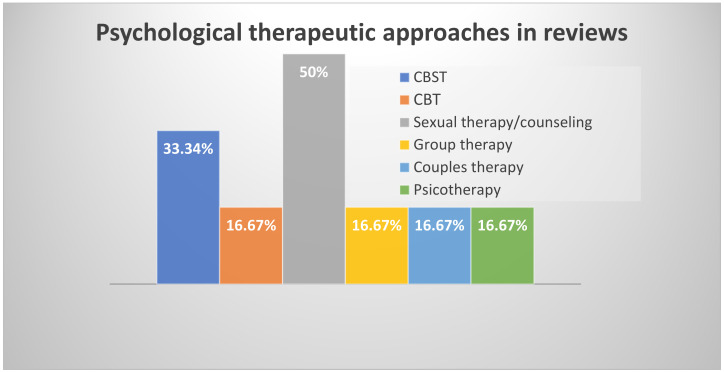
Treatment approaches. Legend: CBST = Cognitive–Behavioral Sexual Therapies; CBT = Cognitive–Behavioral Therapies.

Regarding psychological therapies (Figure 3), where orientation was specified, the overview consisted only of behavioral (*n* = 1) and cognitive–behavioral sexual therapies (*n* = 2). To these were added, without specification of orientation, sexual therapies/counselling (*n* = 3) and group, couple, and psychotherapy therapies (*n* = 1 each).

However, no review’s conclusion described sex education programs (as a source or proposal in the conclusion) or a multidisciplinary course of therapy. Moreover, only half of the reviews reflected hypothetical diagnostic protocols involving a psychologist.

In relation to medical treatments, five of the reviews reported types of pharmacological and surgical treatments, whereas one (16.67%), in addition to these treatments, reported those related to traditional oriental medicine [18].

Table 2 reports other details of the reviews, including the outcomes. In all reviews, a correlation between the presence of SDs, especially EDs, and organic etiology and health status problems was underlined.

**Table 2:** Details of review papers.

Review’s Study Design	Authors	Psychological Therapy	Psychological and Relational Causes	Organic Correlations Highlighted
Narrative review	Sooriyamoorthy et al. [14]	Psychotherapy (orientation not specified); Psychosexual counselling	Anxiety; Depression; Relational components	ED is closely linked to CVD (diabetes mellitus, hyperlipidaemia, and hypertension), among other disorders. Men with erectile dysfunction had 44% more cardiovascular events, 62% more myocardial infarctions, 39% more strokes, and a 25% increased risk of death compared with patients without ED.
Narrative review	Nguyen et al. [13]	Cognitive–behavioral sexual therapy; Psychosexual counselling	Anxiety; Depression; Relational components	ED is correlated with endocrinological risk factors such as diabetes, thyroid disease, excessive soy consumption, and KS (Klinefelter syndrome). For the treatment of ED in young men, oral PDE5Is remain the first-line oral agents used. In addition, surgical and hormonal treatments have been more widely used in the treatment of ED in young men.
Narrative review	Papagiannopoulos et al. [19]	Psychosexual therapy (only regarding possible pre-1970s therapies);	Anxiety (named only for differential with organic causes); Relational components (as a differential between psychogenic and organic ED)	Young men with ED may be at higher risk of future morbidity and mortality: ED may be an indicator of poor general health and CVD. Men with ED should be evaluated for subclinical CVD risk factors.
Narrative review	Ho et al. [10]	Behavioral therapy (for ED)	Depression	Modern medicine is complemented by traditional medicine (e.g., acupuncture, acupressure, yoga, herbal medicine, and spiritual healing): in Malaysia, 65% of men felt that traditional medicines were better than conventional ones.
Scoping review	Rastrelli et al. [16]	Cognitive–behavioral sexual therapy Psychoanalysis (only regarding possible pre-1970s therapies)	Anxiety; Depression; Relational components	ED in young men, even more so than in older men, can be considered a harbinger of CVD. Young men reporting ED risk being dismissed without a specific medical assessment because of the assumption that ED in young men is a self-limiting condition without clinical consequences.
Umbrella review	Allen et al. [15]	Psychosexual therapy; Couple therapy; Group psychotherapy (orientation not specified)	Anxiety (only in relation to the decrease in depression anxiety with alcohol assumption); Depression	Because diabetes, poor diet, obesity, low exercise, and CVD are interconnected, identifying the primary risk factor can be difficult. The most obvious candidate for a primary risk factor is a weakened vascular system.

Legend: ED = erectile dysfunction; CVD = Cardiovascular Disease.

### 3.4. Psychological and Organic Aetiology

A study reported by Nguyen et al. (Figure 2) found that 85.2% of men under 40 years of age had psychogenic ED as the primary etiology, compared with 14.8% who had an organic cause of ED. This finding contrasts starkly with that of the group of patients aged over 40 years, who had a prevalence of 40.7% for psychogenic ED and 59.3% for organic ED. No tests were used for the psychological assessment; only the sexual history account, problem description, and current sexual relationships were assessed [19]. Other studies have lower percentages; for example, a review by the University of California San Francisco showed that 13% of men under age 40 have only psychogenic ED [20]. According to another review, approximately 15–20% of ED cases are of organic origin [21], caused by lifestyle, genetic factors, medical conditions, and the use of certain drugs and medicines. The difference in these results, as we discuss in greater detail in the limits of the review, may be related to a qualitative rather than quantitative approach of the research and differences in the reference samples.

### 3.5. Depression and Anxiety

Approximately two-thirds of the studies in the literature on anxiety and depression referred to people aged over 40 years. Those under 40 showed that poor mental health and depression (tests used the Major Depression Inventory and SF-12 for mental and physical health) were predictive factors for premature ejaculation and ED [21]. A meta-analysis found that the risk of ED increases by 39% in patients with depression, and exposure to ED increases the risk of depression by 192% [22].

The studies also showed that depression appeared to have a somewhat stronger association with present ED than anxiety (symptoms of depression explained 4.2% and symptoms of anxiety 3.0% of the variance in ED), and symptoms of anxiety and depression had a rather strong correlation, suggesting substantial comorbidity between the two (anxiety and depression tested using the Brief Symptom Inventory-18) [21]. Alcohol had a curvilinear effect, in which moderate alcohol intake (linked to decreased performance anxiety and increased sexual desire) appeared beneficial for ED [23].

Excessive preoccupation with physical self-image and, in particular, genital self-image leads to directing most of the mental energy to monitoring the body with consequent impairment of erection [24].

### 3.6. Relational Conditions

Family and couple conflicts were significantly associated with free-floating anxiety and depression symptoms (both tested using the Middlesex Hospital Questionnaire-MHQ) and with a higher risk of ED and hypoactive sexual desire. Nguyen et al. pointed out that the effect of a couple’s relationship on ED is hypothetically different in younger men because of both parties’ lack of experience, fear of emotional involvement, limited privacy, and/or concerns over an unwanted pregnancy [21].

### 3.7. Organic Causes

Regarding organic causes related to sexual dysfunction, we focused on ED and found a variety of disorders or substances consumed, such as neurological diseases, hormonal causes (hypogonadism), trauma to the genital area, diabetes mellitus, metabolic syndrome, and a variety of medications (antidepressants, antihypertensives, antipsychotics, opioids, and recreational drugs) [22]. The correlation between erectile dysfunction and cardiovascular disease (CVD) is the one most frequently highlighted in the reviews. Regarding young patients, we found a deepening of this factor in a study carried out by Yao et al. [25], suggesting that many young men with ED may not have obvious clinical CVD but still have subclinical CVD factors that predispose them to ED [21].

### 3.8. Diagnosis

A problem emerges in the diagnosis related to both an embarrassment of the patient to talk about sexual disorders [18] and, especially in young men, the absence of specific medical assessments because of the false belief that ED is a condition with no other clinical implications [24]. Early diagnosis and treatment are extremely important for ED. They can reduce the emotional stress experienced by patients and their partners [22] and break the vicious circle of sexual failure–loss of confidence and anxiety and/or avoidance, increasing the possibility of future failure [21].

In the diagnostic process, distinguishing between psychological and organic causes of ED and verifying that the patient has erectile dysfunction and not another type of sexual disorder such as premature ejaculation are extremely worthwhile. Elements in the history that point to a psychological etiology include sudden onset of ED (especially if related to a new partner or a major life-changing event), situational ED, normal erections with masturbation or a different partner, the presence of morning erections, and high daily variability in erectile rigidity [22].

### 3.9. Efficacy of Psychological Therapy

As previously mentioned, the most frequently reported orientations in the reviews are behavioral and cognitive–behavioral. One referenced study on the use of sex therapy reported that a positive treatment outcome occurred in 69.4% of patients and was associated with better pretreatment communication and general sexual adjustment, especially the female partner’s interest in and enjoyment of sex, absence of a positive psychiatric history in the female partner, and a couple’s early engagement in homework assignments [22]. Psychological interventions and behavioral changes can lead to improvements at the same level as pharmaceuticals, and the use of phosphodiesterase type 5 inhibitors (PDE5Is) with psychological pathways had better results than single use; however, for both elements, the review indicated difficulty with comparing effect data. [23].

In addition, according to a recent RCT, vardenafil (PDE5I) can improve male sexual function; however, this improvement is only maintained in patients receiving both vardenafil and cognitive behavioral therapy (CBST). In addition, female sexual function and satisfaction improved only in the case of combined vardenafil and CBST therapy, suggesting that a therapy that treats the couple is more effective and has longer-lasting efficacy than the use of a drug that focuses only on ED [24].

Even when obvious psychological problems are not apparent, the involvement of mental health experts can help address associated problems, such as reducing performance anxiety, promoting adherence to treatment, improving relationship problems, identifying interpersonal conflicts, and setting realistic expectations for the couple [22].

### 3.10. Medical Therapy

Several first-line treatment options are available, including lifestyle modifications, testosterone supplementation, topical anesthetic agents, and PDE5Is [23]. Oral PDE5Is remain the first-line oral agents used to treat ED in young men because of their proven efficacy, good tolerability, and ease of administration. This preference is related to immediacy and ease of outcome; long-term measures and lifestyle changes, such as weight reduction, exercise, smoking cessation, healthy eating habits, and stress reduction, are not well received by patients [18].

In addition, surgical therapies, such as extracavernous injection, penile revascularization, inflatable penile prostheses, hormone supplementation, and stem cell therapies, are available [21]. In Asia, modern medicine is combined with and often substituted for traditional Oriental medicine, which accounts for 30–50% of the total consumption of such medicines [18].

## 4. Discussion and Conclusions

The aim of this study was to analyze, through selected reviews of the last 10 years, the risk factors, treatments, and diagnostic cautions for sexual disorders in men under 40 years of age. The scope of the review was to identify good practices related to multidisciplinary interventions and highlight the focal elements.

Considering the exclusion/inclusion criteria, six reviews were included in the review. As mentioned in the previous section, the literature that has emerged seems to focus more on erectile dysfunction than on other disorders, such as premature ejaculation, low desire, delayed ejaculation, or retrograde ejaculation. In fact, only one review of the database, in addition to the ED, was an investigation into other SDs addressed [26].

Despite the effort to search for reviews describing psychological treatment procedures, a prevailing focus on pharmacological and, secondarily, surgical treatments appeared to be highlighted in the reading. This focus also emerged in the analysis of risk factors, for which the focus was on comorbidities with other organic disorders and subclinical conditions, possibly because of the numerous cases of research analyzed in the reviews considered, a lack of attention in the structuring of research outcomes in the psychological area, or an absence of psychologists on the research staff.

Young population. We chose to focus on a young population rather than the over-40s population, on which most of the literature focused, for several reasons. The prevalence of ED in this population might be underestimated because of under-reporting and could be as high as 30%. In the population under the age of 40, unlike the rest of the population, the causes of SDs, especially ED, were mostly psychogenic (85.2% had psychogenic ED as the primary etiology, compared with the group of patients aged over 40 years, who had a prevalence of 59.3% of organic ED). Furthermore, men with more sexual experience (often coinciding with older age) had ED less frequently, possibly suggesting a positive influence of sexual experience and self-confidence. Anxiety may be involved in the pathogenesis of ED, often at the beginning of the sexual life [21].

Recent studies have shown an increase in the incidence of ED in men under 40 years of age, and this trend is probably underestimated because of under-reporting by younger patients [23]. This population, especially adolescents, could be unlikely to present themselves independently to psychological and medical diagnoses for reasons of shame, lack of sexual education, and economic dependence, also because the tendency is to underestimate the problem if one has a disorder without a physical/biological matrix. Therefore, involving them in screening, public information, and psycho-education activities could be necessary because the period of adolescence coincides with the period of awareness of one’s sexual identity and orientation. However, in many countries, including Italy, no standardized education exists in this regard. Worth noting is that none of the reviews included sex education or population screening scenarios. Alternatively, a risk might exist that the disorder will ‘crystallize’, and we will end up with patients, as is often the case, who face a sexological treatment at the time they are trying to have children; therefore, they will face greater difficulty in their treatment because of anxiety, depression, and avoidance of sexuality caused by ED.

Risk factors and therapies. The psychogenic causes of ED include depression, anxiety, and partner-related difficulties; they can not only be the cause, but also the effect of DS [21]. Organic causes may include metabolic syndromes, neurological diseases, hormonal causes (e.g., hypogonadism and thyroid), trauma, use of drugs and substances of abuse, and CVD [22]. Psychological therapy, which the literature mainly indicates as cognitive–behavioral, is also recommended for organic causes, and the use of PDE5 is recommended because improvements have been observed that have been maintained for longer and that have affected the sexual well-being of the partner. Within the therapy, ‘normalization’ could be very important: patients may modify their behaviour to avoid intimate environments or abstain from sexual encounters. This avoidance could be interrupted by reassuring the patient that ED is very common in the male population and by pointing out that many effective treatments are available with minimal side effects [21]. Of course, as addressed in this review, a stronger scientific focus in the literature on medical interventions should still be considered.

Multidisciplinary protocols. No review described a multidisciplinary therapeutic procedure in its conclusion. Moreover, only half of the reviews presented hypothetical diagnostic protocols involving a psychologist. This lack and the presence of a psychologist only as a residual figure in the diagnosis and treatment process seems to contradict the multidisciplinary nature of these disorders. A multidisciplinary protocol with a psychologist trained in psychosexology would improve not only the discrimination of disorders between organic and psychogenic, but also treatment effectiveness. Addressing intrapsychic and relational risk factors could be essential to the screening and treatment of young men with ED, even more so in young men, for whom emotional distress might affect them, even more than it does adult patients, given both the inexperience of the young man and their need to experiment with their sexual identity. Moreover, psychological consequences of ED almost always exist in terms of marital and relationship problems, loss of self-esteem, shame, anxiety, and depression, among others. Erectile dysfunction can cause considerable emotional damage to the patient and his partner, as well as have a significant impact on quality of life [22]. Protocols regarding medical correlations should also consider that young men with ED may be at a higher risk of future morbidity and mortality, indicating that ED may signal poor overall health, which is even more pressing with respect to CVD risk [27].

*Limitation*. Within the work of the inclusion and exclusion of the reviews, as well as the analysis of the reviews, little interest in the literature for the psychological implications and treatment of male sexual disorders seemed to emerge. In the reviews, psychological aspects are often only mentioned without in-depth analysis. This aspect can also be seen in the PRISMA flowchart, in which the passage from 106 reviews (90 excluding duplicates) to the final six is described.

In these studies, heterogeneity in the data on the percentage of EDs in the population seems to exist (e.g., the prevalence of ED in Asia varies between 9% and 73%), a factor that results from the difference in the age and type of populations analyzed and the screening methods using different items with psychological factors not tested but deduced from the patient’s accounts. Research often based on qualitative rather than quantitative instruments, especially in the psychological dimension, has led to conflicting data that are difficult to compare.

Even when indications of risk factors and psychological effects exist, studies on these factors were not reported in all of the reviews. When such studies were present, they were summarized, resulting in having to go to the primary sources of the reviews for specific data. In some cases, the aspects of anxiety, depression, and relational conflicts, despite the fact that the reference reviews focused on the under-40 age group, had a totally or partly over-40 sample. In general, all reviews seemed to report a clear prevalence of medical information and an approach to the detriment of psychological information. This gap in the literature could only be overcome at the source by providing for a multidisciplinary protocol, with significant involvement of the psychologist in the prevention, screening, diagnosis, and treatment phases.

## Figures and Tables

**Table 1:** Characteristics of reviews.

Characteristics of Articles/Studies		Number	%
Platform Publication: *Journal* (=)	Asian journal of andrology	2	33.33%
	Sexual medicine reviews	1	16.67%
	StatPearls	1	16.67%
	The journal of sexual medicine	1	16.67%
	Translational andrology and urology	1	16.67%
First Author’ Country	Australia	1	16.67%
	Italy	1	16.67%
	Malaysia	1	16.67%
	UK	1	16.67%
	USA	2	33.33%
Publication or posted date	2011	1	16.67%
	2015	1	16.67%
	2017	2	33.33%
	2019	1	16.67%
	2021	1	16.67%
Type of review	Narrative	4	66.67%
	Scoping	1	16.67%
	Umbrella	1	16.67%
Psychological and Relational conditions	Anxiety	5	83.33%
	Depression	5	83.33%
	Relational conditions	4	66.67%
Presence of psychological tests in the reference articles	Reviews reporting tests both for depression and anxiety	2	33.34%
	Reviews reporting tests for depression	2	33.34%
	Review without psychological testing	2	33.34%
Psychological therapies currently used	Behavioral therapy	1	16.67%
	Cognitive–behavioral sexual therapy;	2	33.34%
	Couple therapy (*orientation not specified*);	1	16.67%
	Group Psychotherapy (*orientation not specified*);	1	16.67%
	Psychosexual counselling and therapy (*orientation not specified*);	3	50%
	Psychotherapy (*orientation not specified*);	1	16.67%
Description of sex education programs	Yes	0	0%
	No	6	100%
Hypotheses of multidisciplinary diagnosis protocols.	Yes	3	50%
	No	3	50%
Hypotheses of multidisciplinary therapy protocols.	Yes	0	0%
	No	6	100%
Medical treatments reported	Pharmacological and surgical	5	83.33%
	Traditional oriental medicine (in addition to others)	1	16.67%
Sexual Disorder	Erectile Dysfunction	5	83.33%
	Sexual Dysfunctions (ED, premature ejaculation, and hypogonadism)	1	16.67%

## Data Availability

Not applicable.

## References

[1] Aytaç I.A., McKinlay J.B., Krane R.J. (1999). The likely worldwide increase in erectile dysfunction between 1995 and 2025 and some possible policy consequences. BJU Int..

[2] Liu Q., Zhang Y., Wang J., Li S., Cheng Y., Guo J., Tang Y., Zeng H., Zhu Z. (2018). Erectile Dysfunction and Depression: A Systematic Review and Meta-Analysis. J. Sex. Med..

[3] Totaro M., Dimarakis S., Castellini C., D’Andrea S., Parisi A., D’Amato F., Tienforti D., Palazzi S., Baroni M.G., Francavilla S. (2021). Erectile dysfunction in hyperuricemia: A prevalence meta-analysis and meta-regression study. Andrology.

[4] Corona G., Petrone L., Mannucci E., Mansani R., Balercia G., Krausz C., Giommi R., Forti G., Maggi M. (2005). Difficulties in achieving vs maintaining erection: Organic, psychogenic and relational determinants. Int. J. Impot. Res..

[5] Kaplan H.S. (1979). Disorders of Sexual Desire.

[6] Kar S.K., Choudhury A., Singh A.P. (2015). Understanding normal development of adolescent sexuality: A bumpy ride. J. Hum. Reprod. Sci..

[7] Akre C., Berchtold A., Gmel G., Suris J.-C. (2014). The Evolution of Sexual Dysfunction in Young Men Aged 18–25 Years. J. Adolesc. Health.

[8] Boddi V., Fanni E., Castellini G., Fisher A.D., Corona G., Maggi M. (2015). Conflicts Within the Family and Within the Couple as Contextual Factors in the Determinism of Male Sexual Dysfunction. J. Sex. Med..

[9] Hawton K., Catalan J., Fagg J. (1992). Sex therapy for erectile dysfunction: Characteristics of couples, treatment outcome, and prognostic factors. Arch. Sex. Behav..

[10] Manfredi C., Otero J.R., Djinovic R. (2021). Penile girth enhancement procedures for aesthetic purposes. Int. J. Impot. Res..

[11] Chen L., Shi G.-R., Huang D.-D., Li Y., Ma C.-C., Shi M., Su B.-X. (2019). Male sexual dysfunction: A review of literature on its pathological mechanisms, potential risk factors, and herbal drug intervention. Biomed. Pharmacother..

[12] Jern P., Gunst A., Sandnabba K., Santtila P. (2012). Are Early and Current Erectile Problems Associated with Anxiety and Depression in Young Men? A Retrospective Self-Report Study. J. Sex Marital Ther..

[13] Romero-Otero J., Manfredi C., Ralph D., Osmonov D., Verze P., Castiglione F., Serefoglu E.C., Bozzini G., García-Gómez B. (2020). Non-invasive and surgical penile enhancement interventions for aesthetic or therapeutic purposes: A systematic review. Br. J. Urol..

[14] Rosen R., Catania J., Lue T., Althof S., Henne J., Hellstrom W., Levine L. (2008). Impact of Peyronie’s Disease on Sexual and Psychosocial Functioning: Qualitative Findings in Patients and Controls. J. Sex. Med..

[15] Low W.-Y., Khoo E.-M., Tan H.-M., Hew F.-L., Teoh S.-H. (2006). Depression, hormonal status and erectile dysfunction in the aging male: Results from a community study in Malaysia. J. Men’s Health Gend..

[16] Tricco A.C., Lillie E., Zarin W., O’Brien K.K., Colquhoun H., Levac D., Moher D., Peters M.D.J., Horsley T., Weeks L. (2018). PRISMA Extension for Scoping Reviews (PRISMA-ScR): Checklist and Explanation. Ann. Intern. Med..

[17] Haddaway N.R., Page M.J., Pritchard C.C., McGuinness L.A. (2022). *Prisma2020*: An R package and Shiny app for producing PRISMA 2020-compliant flow diagrams, with interactivity for optimised digital transparency and Open Synthesis. Campbell Syst. Rev..

[18] Ho C.C., Singam P., Hong G.E., Zainuddin Z.M. (2011). Male sexual dysfunction in Asia. Asian J. Androl..

[19] Caskurlu T., Tascı A.I., Resim S., Sahinkanat T., Ergenekon E. (2004). The etiology of erectile dysfunction and contributing factors in different age groups in Turkey. Int. J. Urol..

[20] Donatucci C.F., Lue T.F. (1993). Erectile dysfunction in men under 40: Etiology and treatment choice. Int. J. Impot. Res..

[21] Nguyen H.M.T., Gabrielson A.T., Hellstrom W.J. (2017). Erectile Dysfunction in Young Men—A Review of the Prevalence and Risk Factors. Sex. Med. Rev..

[22] Sooriyamoorthy T., Leslie S. (2021). Erectile Dysfunction.

[23] Allen M.S., Walter E.E. (2019). Erectile Dysfunction: An Umbrella Review of Meta-Analyses of Risk-Factors, Treatment, and Prevalence Outcomes. J. Sex. Med..

[24] Rastrelli G., Maggi M. (2017). Erectile dysfunction in fit and healthy young men: Psychological or pathological?. Transl. Androl. Urol..

[25] Yao F., Huang Y., Zhang Y., Dong Y., Ma H., Deng C., Lin H., Liu D., Lu K. (2012). Subclinical endothelial dysfunction and low-grade inflammation play roles in the development of erectile dysfunction in young men with low risk of coronary heart disease. Int. J. Androl..

[26] Leibium S.R., Rosen R.C. (2004). Principi e Pratica di Terapia Sessuale.

[27] Papagiannopoulos D., Nehra A., Khare N. (2015). Evaluation of young men with organic erectile dysfunction. Asian J. Androl..

